# Green synthesis of protein capped silver nanoparticles from phytopathogenic fungus *Macrophomina phaseolina* (Tassi) Goid with antimicrobial properties against multidrug-resistant bacteria

**DOI:** 10.1186/1556-276X-9-365

**Published:** 2014-07-26

**Authors:** Supriyo Chowdhury, Arpita Basu, Surekha Kundu

**Affiliations:** 1 Molecular and Applied Mycology and Plant Pathology Laboratory, Department of Botany, University of Calcutta, 35, Ballygunge Circular Road, Kolkata 700019, India

**Keywords:** Green synthesis, *Macrophomina phaseolina*, Silver nanoparticles, Antimicrobial, Capping, DNA fragmentation

## Abstract

In recent years, green synthesis of nanoparticles, i.e., synthesizing nanoparticles using biological sources like bacteria, algae, fungus, or plant extracts have attracted much attention due to its environment-friendly and economic aspects. The present study demonstrates an eco-friendly and low-cost method of biosynthesis of silver nanoparticles using cell-free filtrate of phytopathogenic fungus *Macrophomina phaseolina*. UV-visible spectrum showed a peak at 450 nm corresponding to the plasmon absorbance of silver nanoparticles. Scanning electron microscopy (SEM), atomic force microscopy (AFM), and transmission electron microscopy (TEM) revealed the presence of spherical silver nanoparticles of the size range 5 to 40 nm, most of these being 16 to 20 nm in diameter. X-ray diffraction (XRD) spectrum of the nanoparticles exhibited 2*θ* values corresponding to silver nanoparticles. These nanoparticles were found to be naturally protein coated. SDS-PAGE analysis showed the presence of an 85-kDa protein band responsible for capping and stabilization of the silver nanoparticles. Antimicrobial activities of the silver nanoparticles against human as well as plant pathogenic multidrug-resistant bacteria were assayed. The particles showed inhibitory effect on the growth kinetics of human and plant bacteria. Furthermore, the genotoxic potential of the silver nanoparticles with increasing concentrations was evaluated by DNA fragmentation studies using plasmid DNA.

## Background

The production, manipulation, and application of nanoscale particles, usually ranging from 1 to 100 nanometers (nm), is an emerging area of science and technology today [1]. Synthesis of noble metal nanoparticles for applications in catalysis, electronics, optics, environmental science, and biotechnology is an area of constant interest [2]. Generally, metal nanoparticles can be prepared and stabilized by physical and chemical methods. Studies have shown that the size, morphology, stability, and physicochemical properties of the metal nanoparticles are strongly influenced by the experimental conditions, the kinetics of interaction of metal ions with reducing agents, and adsorption processes of stabilizing agent with metal nanoparticles [3]. Chemical approaches, such as chemical reduction, electrochemical techniques, and photochemical reduction, are most widely used [2]. Recently, different solvothermal [4] and hydrothermal [5] approaches are employed for inorganic synthesis of nanoparticles. Chemical reduction is the most frequently applied method for the preparation of silver nanoparticles as stable, colloidal dispersions in water or organic solvents [6]. However, several harmful chemical by-products, metallic aerosol, irradiation, etc. are commonly produced during chemical synthesis processes. These, along with the facts that these processes are expensive, time consuming, and typically done on small laboratory scale, render these methods less suitable for large-scale production [7-9]. The approach for production of nanoparticles therefore should be nontoxic, environmentally harmless, as well as cost effective [1].

For green synthesis of nanoparticles, bio-extracts from diverse group of microorganisms act as a reducing and sometimes as a capping agent in nanoparticle synthesis, ranging from algae [10] to bacteria [11] and also from fungi [12]. For large-scale synthesis of nanoparticles in bioreactors, filamentous fungi are better agents for biomass production in comparison to algae and bacteria, since fungal mycelial mat can withstand flow pressure, agitation, and other conditions in the bioreactors [12]. Extracellular secretion of reductive proteins aids in extracellular synthesis of silver nanoparticles avoiding unnecessary cellular interference, and therefore, it is suitable for direct use in various applications. There are reports of mycosynthesis of silver nanoparticles using phytopathogenic fungi like *Fusarium acuminatum*[13], *Aspergillus flavus*[14], *Alternaria alternata*[15], *Coriolus versicolor*[16], *Penicillium fellutanum*[17], and *Fusarium semitectum*[18]. Some fungi investigated were found to be capable of both extra- and intracellular biosynthesis of Ag-NPs having different particle sizes and shapes, but extracellular production of nanoparticles is more desirable from the point of view of easy isolation.

Nanoparticles have some unique size- and shape-dependent physical and optical properties [19]. These unique characters are often responsible for their toxicity to various kinds of microbes such as bacteria, fungi, and also cancerous cells [20-22]. Hence, studies are going on regarding their utility in the diagnosis as well as treatment of different kinds of diseases [23,24]. In this regard, the presence of protein capping material is advantageous because this acts as the anchoring layer for drug or genetic materials to be transported into human cells [25]. The presence of a nontoxic protein cap also increases uptake and retention inside human cells [26].

The present study deals with the extracellular biosynthesis of silver nanoparticles, using cell-free extract of phytopathogenic soil-borne fungus *Macrophomina phaseolina* (Tassi) Goid, the causal organism of charcoal rot disease of about 500 agronomical important crops all over the world [27]. It describes not only a new method of green synthesis of silver nanoparticles but also their physical attributes, antibacterial activity against human and plant pathogenic multidrug-resistant bacteria, the inhibitory effect on the growth kinetics of microbes, the capping material around the silver nanoparticles, as well as their genotoxic effect.

## Methods

*M. phaseolina* was grown in PDA medium at 28°C and was used for the synthesis of silver nanoparticles. The mycelium from solid substrate was inoculated in 50 ml potato dextrose broth (PDB) in 250-ml Erlenmeyer flasks and incubated at 28°C for 5 days. The fully expanded mycelial mat was harvested aseptically and washed with sterile distilled water to remove media components. One gram of washed mat was added to 10 ml of deionized water in a 250-ml Erlenmeyer flask and agitated at 28°C for 72 h in an orbital shaker at 120 rpm. The extract was collected and filtered through Whatman filter paper No. 1 (Whatman, Piscataway, NJ, USA). This cell-free filtrate was used for nanoparticle synthesis. The biosynthesis of silver nanoparticles was done by adding silver nitrate (AgNO_3_) solution to 50-ml cell filtrate to a final concentration of 1 mM in a 250-ml Erlenmeyer flask and agitating in a shaker at 120 rpm at 28°C in the dark for 24, 48, and 72 h. A control set without silver nitrate was simultaneously agitated with experimental set [26]. The silver nanoparticle synthesis was visible by distinct change in coloration of cell filtrate.

The qualitative testing for confirmation of silver nanoparticles was done with UV–vis spectroscopy. One milliliter of sample aliquot from this bio-transformed product was drawn after 24, 48, and 72 h postincubation with silver nitrate solution, and absorbance was recorded by using Hitachi U-2000 spectrophotometer (Hitachi, Ltd., Chiyoda-ku, Japan) range between 350 and 600 nm in order to study the change in light absorption of the solution with increase in color intensity.

About 20 μl of silver nanoparticle solution was spread as a thin film on a glass stub (1 cm × 1 cm) and was vacuum dried. The sample was subjected to scanning electron microscopy using FEI Quanta 200 (FEI, Hillsboro, OR, USA). The average size and shapes of the silver nanoparticles were determined by transmission electron microscopy (TEM). A drop of nanoparticles suspension was placed on a carbon-coated copper grid and was dried under vacuum. Micrographs were obtained in a JEOL JEM 2100 HR transmission electron microscope (JEOL Ltd., Akishima-shi, Japan) with 80- to 200-kV accelerating voltage at 0.23-nm resolution. For atomic force microscopy (AFM) imaging of silver nanoparticles, 10 μl of the nanoparticle suspension was deposited onto a freshly cleaved muscovite Ruby mica sheet (Ruby Mica Co. Ltd., Jharkhand, India) and left to stand for 15 to 30 min. The sample was subsequently dried by using a vacuum dryer and washed with 0.5 ml Milli-Q water (Millipore, Billerica, MA, USA). The sheets were dried again by a vacuum dryer. The size and topography of silver nanoparticles were investigated using atomic force microscope (Model Innova, Bruker AXS Pvt. Ltd, Madison, WI, USA) under tapping mode in which high-resolution surface images were produced. Microfabricated silicon cantilevers of 135-μm length and 8-nm diameter with a nominal spring force constant of 20 to 80 N/m were used. The cantilever resonance frequency was 276 to 318 kHz. The deflection signal is analyzed in the NanoScope IIIa controller (Bruker AXS Pvt. Ltd.), and the images (512 × 512 pixels) were captured with a scan size range of 0.5 and 5 μm.

For X-ray diffraction (XRD) of silver nanoparticles, a thin film of nanoparticle solution was spread evenly on a glass slide and dried by using vacuum dryer. XRD patterns were recorded in a D8 Advance DAVINCI XRD System (Bruker AXS Pvt. Ltd.) operated at a voltage of 40 kV and a current of 40 mA with CuKα radiation (*λ* = 1.54060/1.54443 Å), and the diffracted intensities were recorded from 35° to 80° 2*θ* angles.

The multidrug-resistant strains of *Escherichia coli* (DH5α) and *Agrobacterium tumefaciens* (LBA4404) were prepared according to previous report from our lab [28]. The DH5α-multidrug-resistant (MDR) strain (containing plasmids pUC19 and pZPY112) was selected against antibiotics ampicillin (100 μg/ml) and chloramphenicol (35 μg/ml). LBA4404-MDR containing plasmid pCAMBIA 2301 was selected against antibiotics rifampicin (25 mg/l) and kanamycin (50 mg/l). LB broth/agar were used to culture the bacteria. The disc diffusion method was employed for assaying antimicrobial activities of biosynthesized silver nanoparticles against *E. coli* (DH5α), multidrug-resistant *E. coli* (DH5α-MDR), plant pathogenic bacteria *A. tumefaciens* (LBA4404), and multidrug-resistant *A. tumefaciens* (LBA4404-MDR). One hundred microliters of overnight cultures of each bacterium was spread onto LB agar plates. Concentration of nanoparticles in suspension was calculated according to [27] following the formula $c=\frac{T}{\mathrm{NVA}}$ [where *C* = molar concentration of the nanoparticles solution, *T* = total number of silver atoms added as AgNO_3_ (1 mM), *N* = number of atoms per nanoparticles, *V* = volume of reaction solution in liters, and *A* = Avogadro’s number (6.023 × 1,023)]. The concentration of silver nanoparticles was found to be 51 mg/l. This silver nanoparticle suspension was used in requisite amount for further antimicrobial study. Sterile paper discs of 5-mm diameter with increasing percentage of silver nanoparticles in a total volume of 100 μl (volume made up with sterile double distilled water) were placed on each plate. Ten, 20, 50, 70, and 100% silver nanoparticle solution corresponding to 0.51, 1.02, 2.55, 3.57, and 5.1 μg of silver nanoparticles in 100-μl solution each were placed on the discs. Plates inoculated with *A. tumefaciens* (LBA4404 and LBA4404-MDR) were incubated in 28°C for 48 h, and those inoculated with strains of *E. coli* (DH5α and DH5α-MDR) were kept at 37°C for 12 h. Antimicrobial activity of silver nanoparticles was assessed by measuring inhibition zones around the discs.

In order to observe the effect of the silver nanoparticles on growth kinetics of bacteria, silver nanoparticles were added to the liquid culture of two bacteria, *E. coli* (DH5α) and *A. tumefaciens* (LBA4404). For the initial culture, 7 ml of LB medium was inoculated with 500 μl of overnight grown bacterial culture. This freshly set bacterial culture was supplemented with 2.5 ml of nanoparticle suspension, with concentration of 51 μg/ml. In each of the control sets, 2.5 ml of *Macrophomina* cell filtrate only was added without nanoparticles. The OD values of the mixture was recorded at 600-nm wavelength of visible light at regular time intervals (i.e., 0, 2, 4, 6, 8, 12, and 24 h postinoculation) to plot the growth curves of each of the nanoparticle-treated bacterial cultures and was compared with that of the untreated control.

For isolation of extracellular proteins, about 500 mg of fungal mycelial mat was taken in a microcentrifuge tube, and 500 μl of sterile deionized water was added. The mixture was inverted two to three times for even dispersion of fungal tissue in water. The mixture was gently agitated overnight at 4°C on a shaker. The next day, the slurry was centrifuged at 10,000 rpm for 10 min at 4°C. The cell-free filtrate containing the extracellular proteins was analyzed by one-dimensional SDS-PAGE. In order to isolate the protein(s) bound to the surface of silver nanoparticles, the particles were washed with sterile distilled water and boiled with 1% sodium dodecyl sulfate (SDS) solution for 10 min followed by centrifugation at 8,000 rpm for 10 min for collection of supernatant. The untreated nanoparticles (without boiling in 1% SDS solution) were kept as control. All the other samples were denatured in 2× Laemmli’s sample buffer and boiled for 5 to 10 min, followed by centrifugation at 8,000 rpm at 4°C for 3 min. Electrophoresis was performed in a 12% SDS-polyacrylamide gel using Bio-Rad Mini-PROTEAN gel system (Bio-Rad, Hercules, CA, USA) at a constant voltage of 100 kV for 2 h. Postelectrophoresis, gel was stained with Coomassie Brilliant Blue dye and observed in a gel-imaging system (Chromous Biotech, Bangalore, India).

Genotoxic potential of the silver nanoparticles was tested against plasmid pZPY112 according to [29,30], with minor modifications. Plasmid was isolated from DH5α (containing pZPY112 vector, selected against rifampicin 50 mg/l and chloramphenicol 40 mg/l) by alkaline lysis method. Five micrograms of plasmid was incubated with 0.51, 1.02, 2.55, 3.57, and 5.1 μg of silver nanoparticle (in a total volume of 100 μl solution) in 1 mM Tris (pH = 7.8) for a period of 2 h at 37°C. In control set, cell filtrate was used instead of the nanoparticle solution. Products were run on a 1.5% agarose gel in 1× TAE buffer at 100 V for 45 min and visualized by ethidium bromide staining. Photographs were taken in an UV-transilluminator (Biostep, Jahnsdorf, Germany).

For antimicrobial disc diffusion assay of silver nanoparticles against bacteria, each bar represents mean of three experiments ± standard error of mean (SEM). Differences between treatments (concentration of nanoparticles) in antimicrobial assay were tested using one-way ANOVA (GraphPad Prism, version 5, La Jolla, CA, USA) followed by Tukey’s honestly significant difference (HSD) test, for differences that were significant at 5% probability.

## Results and discussion

### Biosynthesis of silver nanoparticles from cell-free filtrate of *Macrophomina phaseolina*

**Figure 1:**
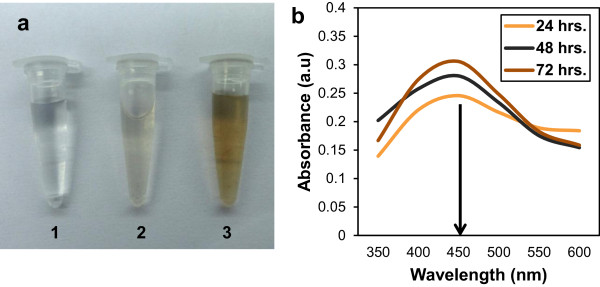
**Synthesis of silver nanoparticles using cell-free filtrate of *Macrophomina phaseolina *and spectroscopic analysis. (a)** Photograph of 1 mM AgNO_3_ solution without cell filtrate (1, control), mycelia-free cell filtrate of *M. phaseolina* (2), and 1 mM AgNO_3_ with cell filtrate after 24-h incubation at 28°C (3). **(b)** UV–vis spectra recorded as a function of time of reaction at 24, 48, and 72 h of incubation of an aqueous solution of 1 mM AgNO_3_ with the *M. phaseolina* cell filtrate showing absorption peak at 450 nm.

The cell-free filtrate of *M. phaseolina* was used for the biosynthesis of the silver nanoparticles as described in methods. Figure 1a shows that AgNO_3_ solution itself is colorless (tube 1). The fungal cell filtrate incubated without AgNO_3_ (tube 2) was also almost colorless. The fungal cell filtrate, after incubation with 1 mM AgNO_3_ (tube 3), underwent a distinct change in its color to brown within 24 h, which indicated the formation of silver nanoparticles due to the conversion of Ag^+^ ions to elemental Ag by extracellular reductase activity of *M. phaseolina* filtrate. The color intensity of the silver nanoparticle solution persisted even after 72 h, which indicated that the particles were well dispersed and stable in the solution. The mycosynthesis of silver nanoparticles involves trapping of Ag + ions at the surface of the fungal cells and the subsequent reduction of the silver ions by the extracellular enzymes like naphthoquinones and anthraquinones present in the fungal system. One earlier study with *Fusarium oxysporum* shows that NADPH-dependent nitrate reductase and shuttle quinine extracellular process are responsible for nanoparticle formation [31]. Extracellular secretion of enzymes is especially advantageous for large-scale nanoparticle synthesis since large quantities of relatively pure enzyme can be obtained, free from other cellular proteins associated with the organism. The nanoparticles thus produced can be easily isolated by filtering from the reaction mix [28].

### UV–vis spectroscopy of the silver nanoparticles

The silver nanoparticles were subjected to spectral analysis by UV–vis spectroscopy. The absorption spectra of nanoparticles showed symmetric single-band absorption with peak maximum at 450 nm for 24, 48, and 72 h of incubation of cell filtrate with AgNO_3_ which steadily increased in intensity as a function of time of reaction without any shift in the peak (Figure 1b). This indicates the presence of silver nanoparticles, showing the longitudinal excitation of surface plasmon, typical of silver nanoparticles.

### Morphological study of the silver nanoparticles with scanning electron microscopy

**Figure 2:**
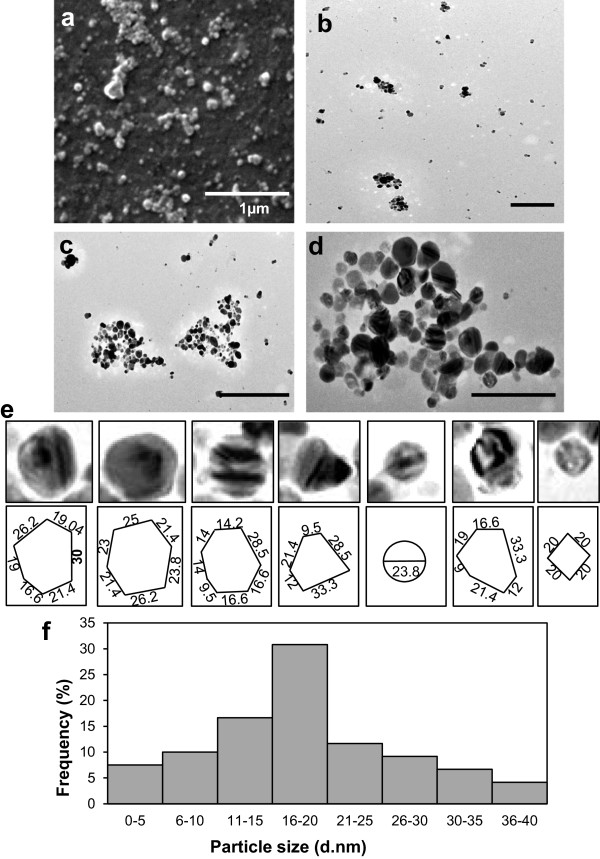
**Electron micrographs of silver nanoparticles. (a)** Scanning electron microscopy micrograph of silver nanoparticles produced with *M. phaseolina* at 50,000 magnification (bar = 1 μm). **(b, c, d)** Transmission electron micrograph of silver nanoparticles at different magnifications (bar = 100 nm). **(e)** Measurement of nanoparticles of different shapes. **(f)** Histogram showing particle size distribution of silver nanoparticles with majority of the particles showing 16 to 20 nm size range.

The morphology (viz shape and size) of the silver nanoparticles studied under scanning electron microscopy (SEM) (magnification × 50,000) revealed that the nanoparticles were mostly spherical in shape and polydisperse in nature (Figure 2a). The nanoparticles were not in direct contact even within the aggregates, indicating stabilization of the nanoparticles by a capping agent.

### Transmission electron microscopy study of silver nanoparticles

Transmission electron microscopy (TEM) micrographs showed that particles are spherical, uniformly distributed without any significant aggregation (Figure 2b,c,d). Some of the nanoparticles showed striations (Figure 2d). The particle size histogram of silver nanoparticles showed that particle size ranges from 3.33 to 40.15 nm with an average size of 17.26 ± 1.87 nm. Frequency distribution observed from histogram showed that majority of particles (30.82%) lie within the range of 16 to 20 nm (Figure 2e). These silver nanoparticles are especially small and polydisperse in nature. This small size range of silver nanoparticles adds to its antibacterial property, since it can easily penetrate bacterial cell membrane and thereafter damage the respiratory chain, affect the DNA, RNA, and division of the cell, and finally lead to cell death [32].

### Morphological study using atomic force microscopy

The shape and size of the silver nanoparticles were further confirmed by atomic force microscopy (AFM). Majority of the particles were symmetrical and spherical in shape and mostly dispersed; although in some places, nanoparticles were found to be in aggregates (Figure S1 in Additional file 1). The graph depicting the profile of the particles under AFM shows most particles were less than 50 nm in height (Figure S1 in Additional file 1).

### X-ray diffraction analysis of silver nanoparticles

**Figure 3:**
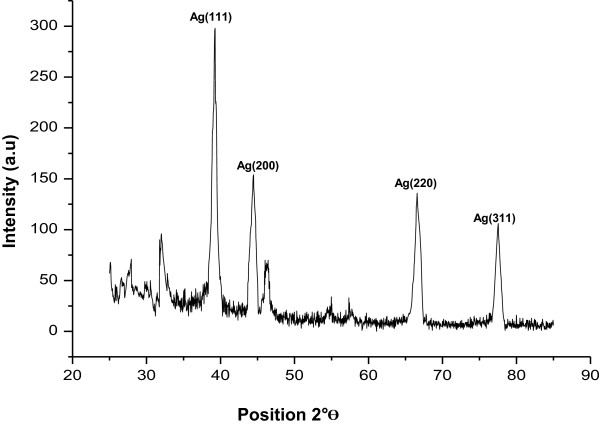
**X-ray diffraction patterns of silver nanoparticles synthesized from cell-free filtrate of *M. phaseolina *** **showing characteristic peaks.**

Due to the crystalline nature of silver nanoparticles, intense X-ray diffraction (XRD) peaks were observed corresponding to the (111), (200), (220), and (311) planes for silver at 2*θ* angles of 38.21°, 47°, 65.27°, and 77.6°, respectively (Figure 3). This was in agreement with the unit cell of the face-centered cubic (fcc) structures (JCPDS file no. 04–0783) with a lattice parameter of *a* = 4.077 A^0^. The exact nature of silver particles formed posttreatment of cell-free filtrate with silver nitrate was best deduced by its XRD spectrum. XRD spectra of pure crystalline silver structures and pure silver nitrate have been published by the Joint Committee on Powder Diffraction Standards (file nos. 04–0783 and 84–0713). A comparison of our XRD spectrum with the standard confirmed that the silver particles formed in our experiment were in the form of nanocrystals. The XRD spectrum in the present study agrees with Bragg’s reflection of silver nanocrystals, similar reported in other literature [15].

### Antimicrobial activity of silver nanoparticles against human and plant pathogenic bacteria and multidrug-resistant bacteria

**Figure 4:**
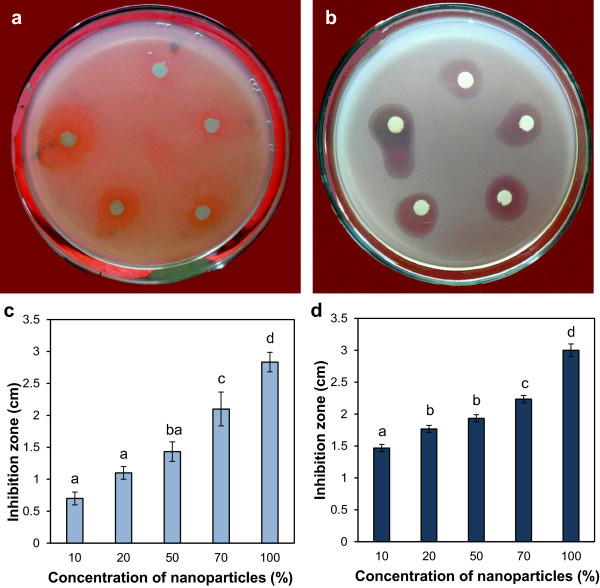
**Antimicrobial effect of silver nanoparticles against normal and multidrug-resistant human bacteria *E*. *coli *by disc diffusion method. (a)** Plate showing increasing inhibition zone of *E. coli* (DH5α) with increasing concentration of nanoparticles: clockwise from top 0.51, 1.02, 2.55, 3.57, and 5.1 μg in a total volume 100 μl in water. **(b)** Plate showing increasing inhibition zone of MDR *E. coli* (DH5α-MDR) with increasing concentration of nanoparticles: clockwise from top 0.51, 1.02, 2.55, 3.57, and 5.1 μg in a total volume 100 μl in water. **(c)** Graph of antimicrobial assay of the nanoparticles on *E. coli* (DH5α ) in which 10, 20, 50, 70, and 100% nanoparticle solution corresponds to 0.51, 1.02, 2.55, 3.57, and 5.1 μg of silver nanoparticles in 100 μl solution, respectively. **(d)** Graph of antimicrobial assay of the silver nanoparticles on MDR *E. coli* (DH5α-MDR). Vertical bars indicate mean of three experiments ± standard error of mean (SEM). Different letters on bars indicate significant differences among treatments (*P* = 0.05).

**Figure 5:**
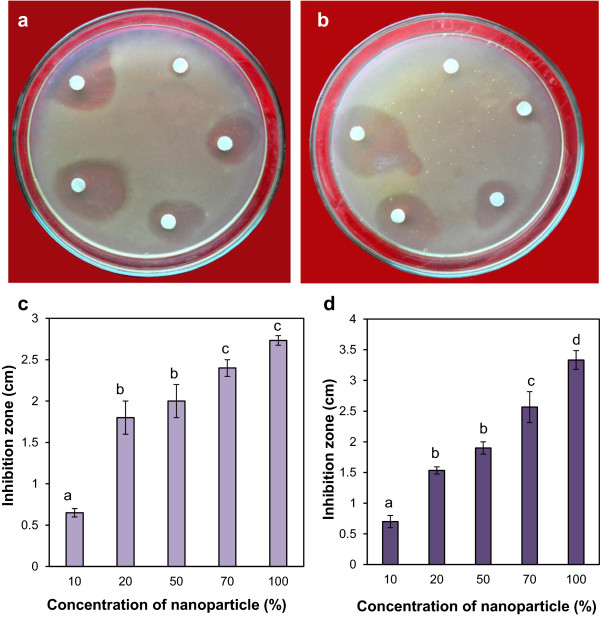
**Antimicrobial effect of silver nanoparticles on normal and multidrug-resistant plant pathogenic bacteria *A. tumefaciens *by disc diffusion method. (a)** Plate showing increasing inhibition zone of *A. tumefaciens* (LBA4404) with increasing concentrations of nanoparticles: clockwise from top 0.51, 1.02, 2.55, 3.57, and 5.1 μg in a total a volume 100 μl in water. **(b)** Plate showing increasing inhibition zone of MDR *A. tumefaciens* (LBA4404-MDR) with increasing concentration of nanoparticles: clockwise from top 0.51, 1.02, 2.55, 3.57, and 5.1 μg in a total volume of 100 μl in water. **(c)** Graph of antimicrobial assay of the nanoparticles on *A. tumefaciens* (LBA4404) in which 10, 20, 50, 70, and 100% nanoparticle solution corresponds to 0.51, 1.02, 2.55, 3.57, and 5.1 μg of silver nanoparticles in 100 μl solution. **(d)** Graph of antimicrobial assay of the silver nanoparticles on MDR *A. tumefaciens* (LBA4404-MDR). Vertical bars indicate mean of three experiments ± standard error of mean (SEM). Different letters on bars indicate significant differences among treatments (*P* = 0.05).

The human bacteria *E. coli* and the plant pathogenic bacteria *A. tumefaciens* were used to assay the antimicrobial activity of the silver nanoparticles. The normal *E. coli* (Figure 4a) as well as the MDR *E. coli* (Figure 4b) plates showed inhibition zones which increased with the increase in concentration of nanoparticles. The graphs of the inhibition zones show nearly similar inhibitory activity of the nanoparticles against the normal and the MDR *E. coli* (Figure 4c,d). Similarly, normal and MDR *A. tumefaciens* plates showed increase in inhibition zones in response to increase in nanoparticle concentration (Figure 5a,b). The graphs of inhibition zone as a function of increasing concentration of nanoparticles (Figure 5c,d) showed similar trend with that of the *E. coli.* In general, *A. tumefaciens* (both LBA4404 and LBA4404 MDR) showed greater sensitivity to the silver nanoparticles than *E. coli* (DH5α) and multidrug-resistant *E. coli* (DH5α-MDR).

All the four microbes tested (DH5α, DH5α-MDR, LBA4404, LBA4404-MDR) against silver nanoparticles were inhibited significantly (*P* = 0.05) in a dose-dependent manner. The antimicrobial activity exhibited by silver nanoparticles is shown in the graph of inhibition zone of four bacteria as a function of increasing concentration of nanoparticles (Figures 4 and 5). In general, both *E. coli* (DH5α) and multidrug-resistant *E. coli* (DH5α-MDR) showed greater sensitivity to silver nanoparticles than *A. tumefaciens* (LBA4404 and LBA4404 MDR). Although, the exact mechanism by which silver nanoparticles act as antimicrobial agent is not fully understood, there are several theories. Silver nanoparticles can anchor onto bacterial cell wall and, with subsequent penetration, perforate the cell membrane (pitting of cell membrane) ultimately leading to cell death [33]. The dissipation of the proton motive force of the membrane in *E. coli* occurs when nanomoles concentration of silver nanoparticles is given [34]. Earlier studies with electron spin resonance spectroscopy revealed that free radicals are produced by silver nanoparticles in contact with bacteria, which damage cell membrane by making it porous, ultimately leading to cell death [31]. Antimicrobial activities of silver nanoparticles from other fungal sources like *F. semitectum*[18] and *Aspergillus niger*[35] gave similar observations. A previous study from our laboratory [28] reported similar antimicrobial activities of silver nanoparticles from *Tricholoma crassum* against human and plant pathogenic bacteria.

### Effect of the silver nanoparticles on the kinetics of microbial growth

**Figure 6:**
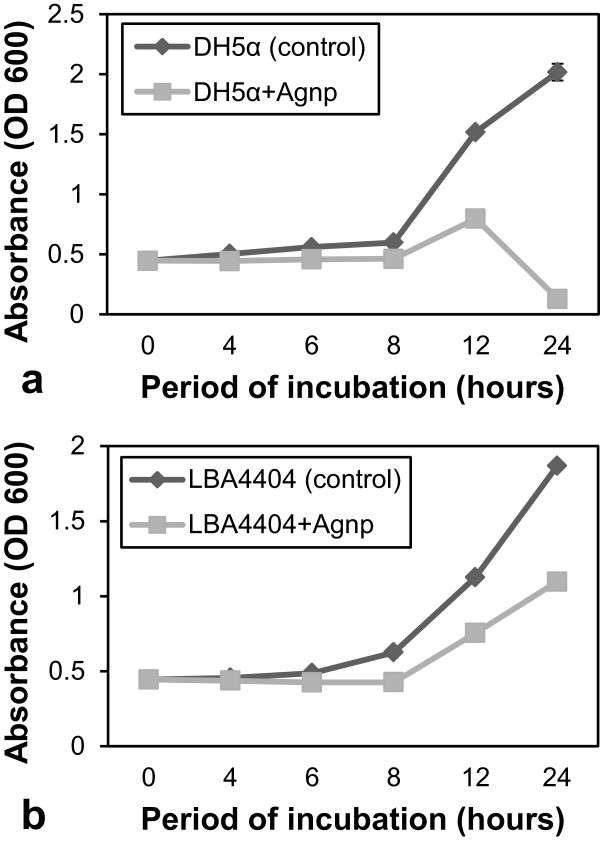
**Inhibitory effect of silver nanoparticles on the growth kinetics of human and plant pathogenic bacteria. (a)** Absorbance data for bacterial growth of plant pathogenic bacteria (*Agrobacterium tumefaciens)* LBA4404 without or with the nanoparticles for 0, 4, 6, 8, 12, and 24 h postinoculation. **(b)** Absorbance data for bacterial growth of human pathogenic bacteria (*E. coli)* DH5α without or with nanoparticles for 0, 4, 6, 8, 12, and 24 h postinoculation showing significant inhibitory effect on the growth kinetics of the bacteria.

The growth kinetics of the bacteria *E. coli* DH5α (Figure 6a) and *A. tumefaciens* LBA4404 (Figure 6b) were clearly suppressed by the addition of the nanoparticles. Growth of both *E. coli* and *A. tumefaciens* showed inhibition of growth within 4 h postinoculation with less optical density readings at all subsequent time points compared to the control. This has been attributed to the reduced growth rate of bacterial cells due to antimicrobial activity of silver nanoparticles.

### Analysis of capping protein around the silver nanoparticles

**Figure 7:**
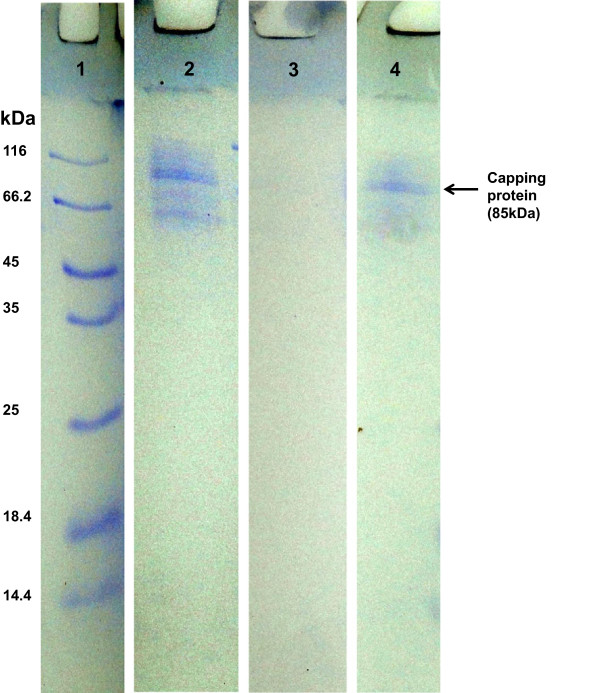
**SDS-PAGE analysis of capping protein around the silver nanoparticles.** Lane 1, molecular size marker; lane 2, extracellular proteins in the cell filtrate; lane 3, nanoparticles loaded without boiling show no protein band; and lane 4, nanoparticles after boiling with 1% SDS loading buffer show a major 85-kDa capping protein.

Sometimes during the biosynthesis process, after the production of silver nanoparticles, reaction is followed by stabilization of nanoparticles by capping agents (i.e., extracellular proteins) from fungal mycelia [16]. To characterize the extracellular fungal proteins associated with the silver nanoparticles, SDS-PAGE was used. Cell filtrate (CF) was isolated by centrifugation from mycelial mat slurry. Protein profiles of cell filtrate clearly showed the presence of several bands of molecular weights between 50 and over 116 kDa (Figure 7, lane 2). Some of these proteins may be responsible for synthesis as well as stability of the silver nanoparticles. Treatment of silver nanoparticles with 1% SDS in boiling water bath for 10 min resulted in detachment of the capping protein(s) from the nanoparticles. When analyzed by SDS-PAGE, the boiled sample showed an intense band of 85 kDa (Figure 7, lane 4) which was not seen when the nanoparticles were not boiled with sample buffer (Figure 7, lane 3). This band is similar to the protein band present in the cell filtrate (Figure 7, lane 2). It is likely that this 85-kDa protein acts as a capping material and confers stability to the silver nanoparticles. Detection of extracellular proteins responsible for synthesis and stability of silver nanoparticles were also reported from a few other literatures [14,36]. The presence of natural capping proteins eliminates the postproduction steps of capping which is necessary for most of applications of nanoparticles in the field of medicine.

### Genotoxic effect of silver nanoparticles against plasmid DNA

**Figure 8:**
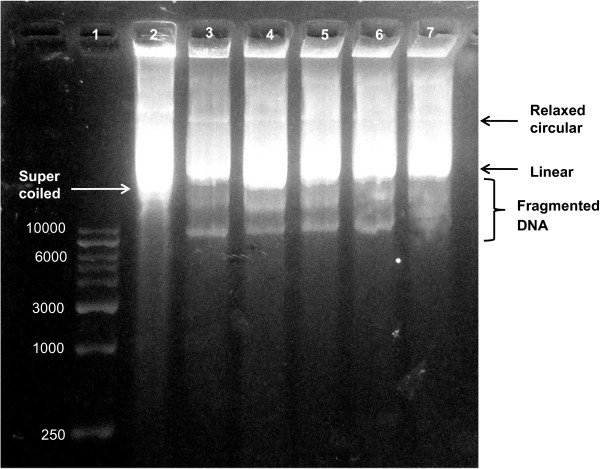
**Agarose gel electrophoresis of plasmid pZPY112 treated with different concentrations of the silver nanoparticles (μg/100 μl).** Lane 1, DNA molecular weight marker. Lane 2, control plasmid without silver nanoparticles showing only supercoiled plasmid band that moves ahead of relaxed circular and linear plasmids. Lane 3, plasmid incubated with 0.51 μg nanoparticles showing disappearance of the supercoiled plasmid band and appearance of relaxed circular and linear plasmid bands along with smaller fragmented DNA. Lane 4, plasmid incubated with 1.02 μg nanoparticles. Lane 5, plasmid incubated with 2.55 μg nanoparticles. Lane 6, plasmid incubated with 3.57 μg nanoparticles showing gradual degradation of the fragmented DNA bands; and lane 7, plasmid incubated with 5.1 μg nanoparticles showing more degradation of DNA.

Agarose gel electrophoresis of plasmid pZPY112 treated with different concentrations of silver nanoparticles showed a dose-dependent induction of DNA strand break, characterized by increased degradation of supercoiled form to relaxed circle to linear forms with increase in concentration of nanoparticles used (Figure 8). DNA strand scission induced by silver nanoparticle leads to gradual degradation in the amount of both linear and supercoiled DNA and appearance of extra bands lower in the gel which are the resultant fragmented DNA (Figure 8). Besides their antimicrobial activity, silver nanoparticles have been shown to be potentially genotoxic by in vivo and in vitro assays [37]. In the present study, the genotoxicity exhibited by silver nanoparticles was demonstrated by degradation of plasmid posttreatment even with low concentrations of the nanoparticles. Such genotoxic activities of nanoparticles were reported earlier in case of carbon nanotubes [38] where degree of DNA degradation was directly proportional to the concentration of nanoparticles. A proposed mechanism of DNA damage is through generation of singlet oxygen as reported in the case of copper nanoparticles [30].

## Conclusions

In this study, phytopathogenic fungus *M. phaseolina* (Tassi) Goid was used for the first time for the extracellular biosynthesis of silver nanoparticles by bioreduction of aqueous Ag + ion. SEM, TEM, and AFM were used to study the morphology, concentration, and size of biosynthesized nanoparticles. The silver nanoparticles exhibited distinct antimicrobial property on multidrug-resistant human and plant pathogenic bacteria. An 85-kDa protein present in the extracellular solution was responsible for synthesis and capping of nanoparticles. This eco-friendly, cost-effective extracellular biosynthesis of naturally protein-capped silver nanoparticles with potent antimicrobial activities from the phytopathogenic fungus has the potential to be utilized on a large scale for widespread industrial or medical application.

## Competing interests

The authors declare that they have no competing interest.

## Authors’ contribution

SK conceptualized and designed all the experiments and acquired funding. SC synthesized nanoparticles, did characterization studies, and interpreted and discussed the results. AB performed the antimicrobial studies. SC and SK drafted the manuscript. All authors read and approved the final manuscript.

## Supplementary Material

Additional file 1: Figure S1 Atomic force microscopy of the silver nanoparticles. (a) AFM images showing top view of the silver nanoparticles. (b) AFM showing three-dimensional view of the nanoparticles. (c) Graphical profile for heights of the nanoparticles based on AFM image. Click here for file
