# Supplementary figures and images for: Green synthesis of protein capped silver nanoparticles from phytopathogenic fungus Macrophomina phaseolina (Tassi) Goid with antimicrobial properties against multidrug-resistant bacteria
Source: Nanoscale Res Lett. 2014 Jul 26;9(1):365. doi: 10.1186/1556-276X-9-365 (PMC4114801; doi:10.1186/1556-276X-9-365)

## Slide 1
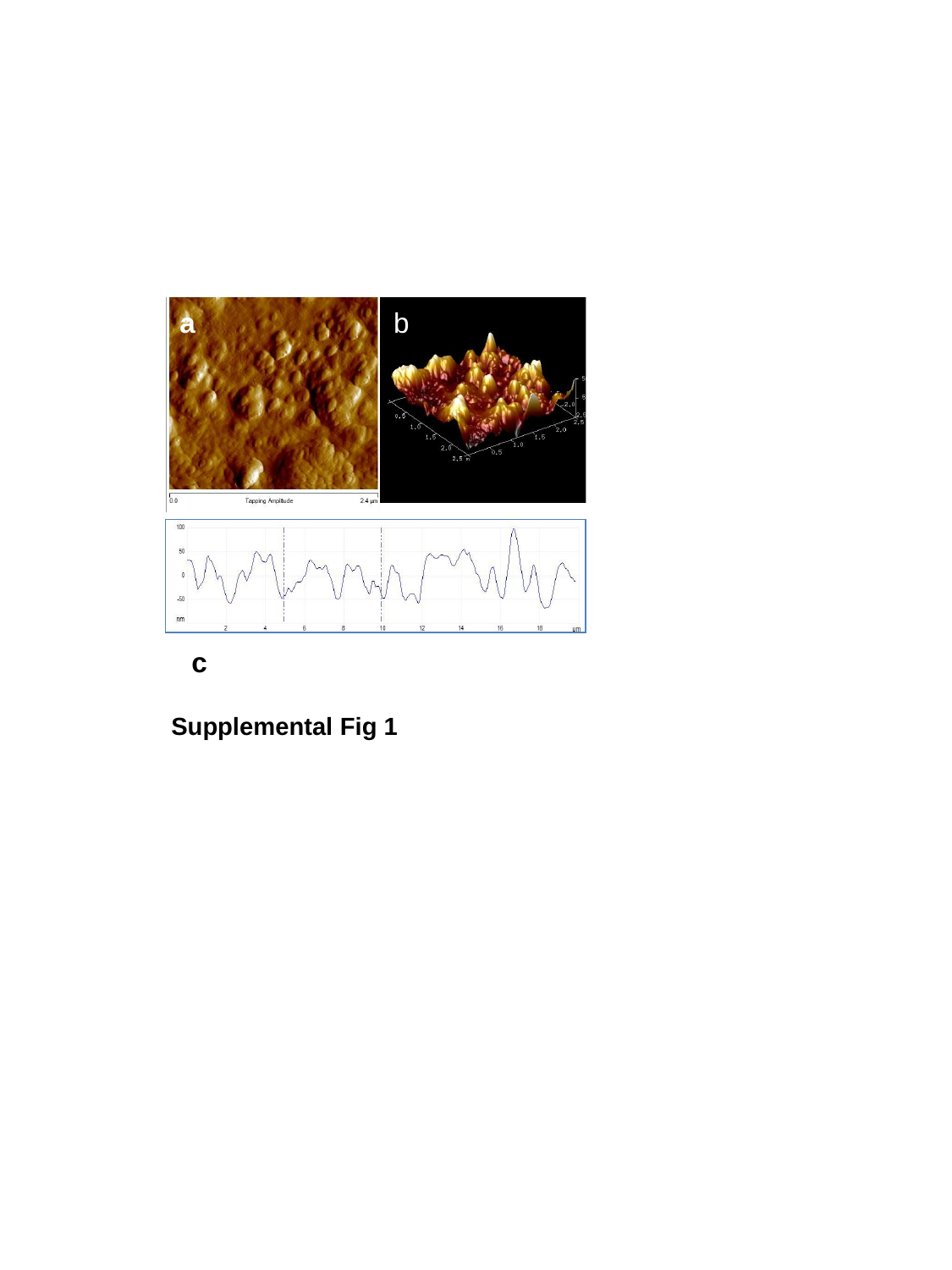

a
b
c
Supplemental Fig 1

Supplement: Additional file 1: Figure S1 — Atomic force microscopy of the silver nanoparticles. (a) AFM images showing top view of the silver nanoparticles. (b) AFM showing three-dimensional view of the nanoparticles. (c) Graphical profile for heights of the nanoparticles based on AFM image. [file 1556-276X-9-365-S1.ppt]
